# Supplementary material for: The hippocampus supports deliberation during value-based decisions
Source: eLife. 2019 Jul 3;8:e46080. doi: 10.7554/eLife.46080 (PMC6693920; doi:10.7554/eLife.46080)
Supplement: Transparent reporting form [file elife-46080-transrepform.docx]

***eLife’s* transparent reporting form**

We encourage authors to provide detailed information *within their submission* to facilitate the interpretation and replication of experiments. Authors can upload supporting documentation to indicate the use of appropriate reporting guidelines for health-related research (see [EQUATOR Network](http://www.equator-network.org/%20)), life science research (see the [BioSharing Information Resource](https://biosharing.org/)), or the [ARRIVE guidelines](http://www.plosbiology.org/article/info:doi/10.1371/journal.pbio.1000412) for reporting work involving animal research. Where applicable, authors should refer to any relevant reporting standards documents in this form.

If you have any questions, please consult our Journal Policies and/or contact us: [editorial@elifesciences.org](mailto:editorial@elifesciences.org).

**Sample-size estimation**

- You should state whether an appropriate sample size was computed when the study was being designed
- You should state the statistical method of sample size computation and any required assumptions
- If no explicit power analysis was used, you should describe how you decided what sample (replicate) size (number) to use

Please outline where this information can be found within the submission (e.g., sections or figure legends), or explain why this information doesn’t apply to your submission:

No statistical method was employed to pre-determine the sample size. The sample size we chose is similar to that used in previous publications. This information is stated under the “Human subjects” section of Materials and Methods (page 24, lines 524-547 in the revised manuscript).

**Replicates**

- You should report how often each experiment was performed
- You should include a definition of biological versus technical replication
- The data obtained should be provided and sufficient information should be provided to indicate the number of independent biological and/or technical replicates
- If you encountered any outliers, you should describe how these were handled
- Criteria for exclusion/inclusion of data should be clearly stated
- High-throughput sequence data should be uploaded before submission, with a private link for reviewers provided (these are available from both GEO and ArrayExpress)

Please outline where this information can be found within the submission (e.g., sections or figure legends), or explain why this information doesn’t apply to your submission:

The fMRI study (experiment 1) was conducted once on 33 participants. Three participants were excluded from analysis of data from this experiment due to excessive motion during the MRI scan. This information is included in the “Human subjects” section of Methods and Material (page 24, lines 525-535 in the revised manuscript).

The patient study (experiment 2) was conducted once on 8 amnesic patients and 16 age-, education- and verbal IQ-matched healthy controls. Two patients and two healthy controls were excluded from analysis. This information is included in the “Human subjects” section of Materials and Methods (page 24-25, lines 538-564).

**Statistical reporting**

- Statistical analysis methods should be described and justified
- Raw data should be presented in figures whenever informative to do so (typically when N per group is less than 10)
- For each experiment, you should identify the statistical tests used, exact values of N, definitions of center, methods of multiple test correction, and dispersion and precision measures (e.g., mean, median, SD, SEM, confidence intervals; and, for the major substantive results, a measure of effect size (e.g., Pearson's r, Cohen's d)
- Report exact p-values wherever possible alongside the summary statistics and 95% confidence intervals. These should be reported for all key questions and not only when the p-value is less than 0.05.

Please outline where this information can be found within the submission (e.g., sections or figure legends), or explain why this information doesn’t apply to your submission:

Behavioral data analysis methods are detailed in the “Behavioral analysis” section of Materials and Methods (page 31-36 lines 717-848).

fMRI analysis methods are detailed in the “Imaging analysis” section of Materials and Methods (page 36-43 lines 851-1013).

Averages and standard errors of the mean are presented in Figures 2, 5, Figure 2—figure supplement 1, Figure 5—figure supplement 1, and Figure 5—figure supplement 4. Exact p-values are found in the results section and in Appendix 1.

(For large datasets, or papers with a very large number of statistical tests, you may upload a single table file with tests, Ns, etc., with reference to sections in the manuscript.)

**Group allocation**

- Indicate how samples were allocated into experimental groups (in the case of clinical studies, please specify allocation to treatment method); if randomization was used, please also state if restricted randomization was applied
- Indicate if masking was used during group allocation, data collection and/or data analysis

Please outline where this information can be found within the submission (e.g., sections or figure legends), or explain why this information doesn’t apply to your submission:

In experiment 1 participants were recruited from the community on Columbia University’s campus and surrounding area in New York City, all participants in this experiment were placed in the same group and participated in the same tasks. The order in which tasks proceeded and the button-color mapping in the perceptual decision-making task was counterbalanced across participants.

In experiment 2, patients were well characterized clinically; they were diagnosed with amnesia due to damage to the hippocampus and surrounding medial temporal lobe and have been followed by clinicians at the memory disorders research center at the VA Boston Healthcare System. Healthy controls were recruited from the Boston area community to match the patient group in age, education level, and verbal IQ.

**Additional data files (“source data”)**

- We encourage you to upload relevant additional data files, such as numerical data that are represented as a graph in a figure, or as a summary table
- Where provided, these should be in the most useful format, and they can be uploaded as “Source data” files linked to a main figure or table
- Include model definition files including the full list of parameters used
- Include code used for data analysis (e.g., R, MatLab)
- Avoid stating that data files are “available upon request”

Please indicate the figures or tables for which source data files have been provided:

File Bakkour_eLife_Figure2SourceData2.csv contains the behavioral data (at the trial level) for perceptual decisions across all 30 participants. These data were used to produce Figure 2A and Figure 2—figure supplement 1.

File Bakkour_eLife_Figure2SourceData3.csv contains the behavioral data (at the trial level) for value-based decisions across all 30 participants. These data were used to produce Figure 2B, Figure 2—figure supplement 1, and Figure 5—figure supplement 5.

File Bakkour_eLife_Figure5SourceData2.csv contains the behavioral data (at the trial level) for perceptual decisions across all 6 patients and 14 healthy control participants. These data were used to produce Figure 5A and Figure 5—figure supplement 1.

File Bakkour_eLife_Figure5SourceData3.csv contains the behavioral data (at the trial level) for value-based decisions across all 6 patients and 14 healthy control participants. These data were used to produce Figure 5B, Figure 5—figure supplement 1, and Figure 5—figure supplement 4.

File Bakkour_eLife_Figure2SourceCode.ipynb is an ipython jupyter notebook that contains the code and outputs for the analyses conducted on data from Experiment 1

File Bakkour_eLife_Figure5SourceCode.ipynb is an ipython jupyter notebook that contains the code and outputs for the analyses conducted on data from Experiment 2

Full fMRI statistical maps have been uploaded to neurovault.org. URL links to the maps presented in figures are provided in the figure caption in the revised manuscript. The raw fMRI data will be made public through openfmri.org
